# Can Systematic Drift Rate Variability Replace Random Variability in the Diffusion Decision Model?

**DOI:** 10.1007/s42113-026-00264-0

**Published:** 2026-03-19

**Authors:** Jie Sun, Daniel Feuerriegel, Adam F. Osth

**Affiliations:** Melbourne School of Psychological Sciences, The University of Melbourne, Melbourne, Australia

**Keywords:** Diffusion decision model, Decision-Making, Across-Trial drift rate variability, EEG, Recognition memory

## Abstract

The full diffusion decision model (DDM) assumes that the rate of evidence accumulation varies across trials, which allows the model to account for slow errors and asymptotic accuracy. This across-trial drift rate variability, however, has been criticised for being difficult to estimate and ad hoc. To examine whether random drift rate variability corresponds to meaningful variations in the quality of decision evidence, we assessed whether the model-estimated drift rate variability parameter can be partitioned by trial-level systematic drift rate information. Using a large recognition memory dataset with electroencephalography (EEG) recordings (*n* = 132), we systematically linked drift rate to individual trials using exogenous experimental factors—such as word frequency and study-test lag—along with endogenous factors using EEG data. Using simulations, we first demonstrated that, when slow errors arise solely due to across-trial drift rate variability, the random variability can be well partitioned and replaced by systematic variability on the trial-level. However, for the experimental data, the inclusion of systematic variability resulted in little decrease in the random across-trial drift rate variability parameter. These findings indicate that, while the quality of decision-relevant evidence (and hence drift rate) is expected to vary across trials, other mechanisms that produce slow errors are likely present but not implemented in the full DDM.

**Supplementary Information:**

The online version contains supplementary material available at 10.1007/s42113-026-00264-0.

## Introduction

Cognitive science research commonly attempts to infer processes underlying human cognition based on measurements such as accuracy scores and/or reaction times (RTs) observed in cognitive tasks. While early studies focused on univariate approaches to analyse how these behavioural markers vary across experimental manipulations, models that jointly describe both accuracy scores and RTs provided additional constraints. Evidence accumulation models have gained popularity in psychological research as they provide an unified account of accuracy and RTs observed in decision-making tasks (Brown & Heathcote, 2008; Ratcliff et al., 2016b; Roe et al., 2001; Usher & McClelland, 2001). These models generally assume that decisions are made by gradually sampling evidence until a decision boundary is reached, allowing predictions of both the time taken to reach these boundaries and which boundary is reached, signifying RT and accuracy.

The Diffusion Decision Model (DDM) is among the most extensively utilised evidence accumulation models in cognitive science (Ratcliff, 1978; Ratcliff & McKoon, 2008). Its success in capturing complex patterns of accuracy and RT data can be partly attributed to the incorporation of trial-to-trial variability in model parameters. For instance, Ratcliff (1978) introduced the assumption of across-trial drift rate variability, in which the drift rate—representing the quality of evidence accumulation—varies across each experimental trial and is sampled from a normal distribution. While this assumption enabled the model to capture slow errors and asymptotic accuracy, it has been criticised for being both difficult to estimate and ad hoc.

The original motivation behind drift rate variability is to be a placeholder for systematic variability. Ratcliff (1978) argued that drift rate variability is a plausible because items studied in recognition memory tasks differs in their memory strength. In this investigation, we took the step of replacing the model-estimated, purely random drift rate distributions with systematic variability that varies across items and experimental trials. We expect such replacement to be feasible if the variability estimates can meaningfully account for model predictions of data (i.e., slow errors). While we have demonstrated the feasibility of this replacement with simulation where the drift rate variability was the sole source of slow errors, we found that the inclusion of a large amount of systematic variability did not lead to a large decrease in random drift rate variability with experimental data.

**Fig. 1:**
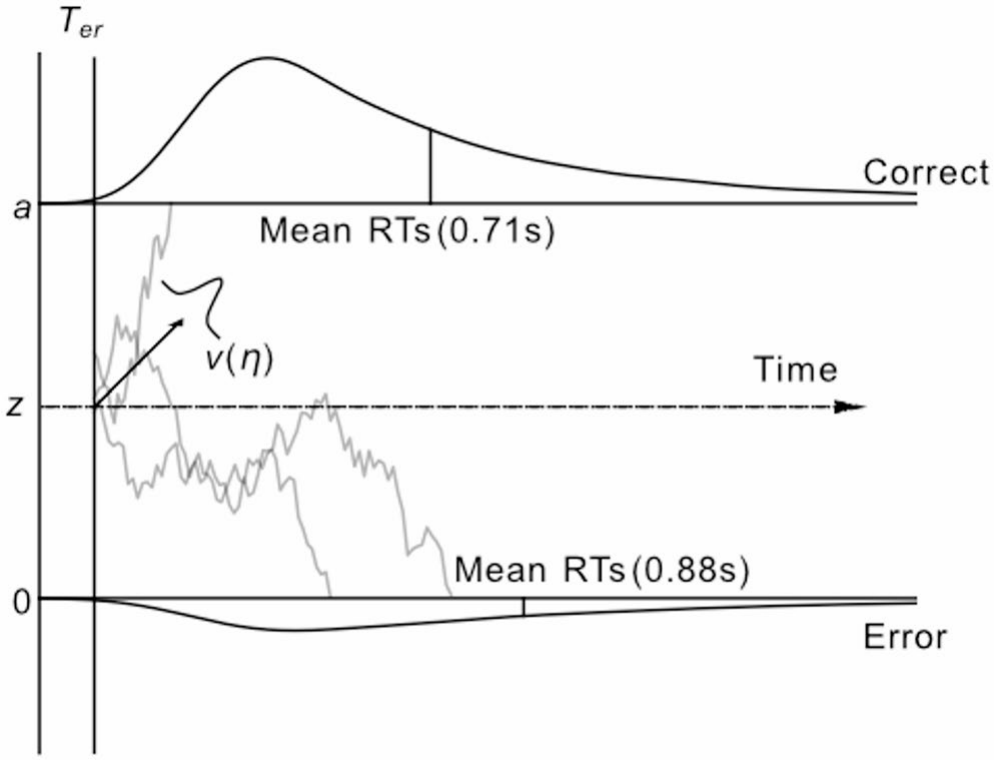
Illustration of the DDM with additional across-trial drift rate variability parameter that predicts slow errors. RT distributions were generated based on simulations. *z* = starting point, *a* = boundary separation, *T*_er_ = non-decision time, *v* = drift rate, *η* = drift rate variability

In its standard form for modelling behaviour in a two-choice task, DDM assumes that decision evidence is continuously sampled from a starting point *z* that could be biased towards one of the two decision boundaries. The model also partitions RTs into decision time and non-decision time *T*_er_, the latter reflecting other processes such as stimulus encoding and motor execution. The separation of boundaries parameter *a* has been interpreted as response caution (Ratcliff et al., 2001), influencing the speed-accuracy trade-off, while the average rate of evidence accumulation (drift rate) reflects the strength of the decision evidence (Fig. 1).

This four-parameter DDM, however, counter-intuitively predict RTs for correct and error responses to be the same; in practice, slower error responses than correct responses have been commonly observed when tasks are difficult and accuracy is emphasised (Luce, 1991; Swensson, 1972). Ratcliff (1978) accounted for this by introducing the assumption that the drift rate varies across trials, following a normal distribution with a mean of *v* and a standard deviation of *η* (Fig. 1, Ratcliff, 1978). When all other parameters are fixed, a higher drift rate in a single trial is associated with a faster RT and a correct response. When there is across-trial variability in the drift rate, trials with higher drift rates are associated with faster RTs and are more likely to be accurate, and therefore produce a smaller proportion of errors. Conversely, trials with lower drift rates will be correspond to slower RTs and will produce a higher proportion of errors. Consequently, RTs will generally be slower in trials with errors. This distributional assumption also enables the model to predict asymptotic accuracy when the decision boundary is raised to infinity; similar performance asymptotes have been observed in the memory literature (McElree & Dosher, 1989). Importantly, the *η* parameters represents random variability in the drift rate, without specifying exactly how or why it fluctuates across trials.

The inclusion of the distributional assumption in Ratcliff (1978) is theoretically grounded in Signal Detection Theory (SDT, Green & Swets, 1966). The motivation comes from the fact that difficulty in the cognitive task typically varies across trials and participants also vary in their degree of attention and encoding quality. Studies have repeatedly shown that evidence contributing to decision (i.e., drift rate) varies from trial-to-trial. Ratcliff et al. (2018) have demonstrated this across a range of perceptual decision-making tasks using a double pass procedure. They included pairs of trials in which the same stimuli were presented, separated by a long interval and many intervening trials. They found that, despite the same stimuli being presented, performance varied to some extent within each pair of trials. They suggested variability in the stimuli characteristics could not fully explain variability in performance, and that other endogenous factors—such as fluctuations in attention—should also be considered as contributors to drift rate variability.

Further, in recognition memory tasks—where participants memorize a list of items and are later instructed to discriminate between previously-encountered (old) versus new items—variability in memory strength for old items has consistently been found to be greater than for new items (Chen et al., 2024; Osth et al., 2017; Starns & Ratcliff, 2014). Memory strength has been considered to drive recognition memory decisions in the signal detection models, with previous-seen items associated with greater memory strength on average than new items. In DDM, memory strength is equivalent to drift rate. Similarly, drift rate variability for previous-seen items were also found to be greater than new items in recognition memory tasks (Starns & Ratcliff, 2014).

Starns (2014) further demonstrated the variability of evidence in a numerosity task. In this task, participants were required to judge whether the number of asterisks presented was above or below 50. In a series of conditions, Starns manipulated the average number of asterisks presented across trials and the variance in the numbers, therefore directly changing the underlying evidence strength distribution across conditions. By fitting the DDM, he found that the relative order of *η* estimates were consistent with orders of the underlying evidence variability.

### Mechanisms for Slow Errors

While these studies have clearly demonstrated evidence for evidence quality varying across trials, it does not necessarily mean that such variation is solely responsible for slow errors. Other mechanisms have been proposed to be responsible for this phenomenon. For example, slow errors could be explained by allowing the parameters of evidence accumulation to vary within trials instead of across trials. For instance, some variants of the DDM include two decision boundaries that gradually shift toward each other over time within a trial (e.g., collapsing boundaries, Bowman et al., 2012; Ditterich, 2006) or within-trial noise that increases over time within a trial (Asadpour et al., 2024). In these models, slower decisions are more prone to reach the error boundary, resulting in slow errors. Alternatively, slow errors can be explained by lateral inhibition as implemented in the Leaky Competing Accumulator (LCA) model (Usher & McClelland, 2001). In this model, evidence accumulates with decay, and accumulators for correct and incorrect choices inhibit each other. When inhibition is stronger than decay, slower responses are more likely to be dominated by the inhibition process (if sufficiently strong), leading to a higher proportion of errors (Yi, 2020). Additionally, introducing a racing structure to evidence accumulation models has also been suggested to produce the slow error pattern (Hawkins & Heathcote, 2021; Tillman et al., 2020), although the effect predicted by such models is small (Smith, 2023).

### The Ad-hoc Criticism

Across-trial variability in diffusion model parameters has also been criticized as being an ad-hoc assumption. Jones and Dzhafarov (2014b) demonstrated that, if there is unlimited flexibility in the underlying distributions of model parameters, a model can account for virtually any pattern of data and become unfalsifiable. While responses to the Jones and Dzhafarov work have emphasized that virtually all work with evidence accumulation models does not use such unconstrained distributions (Heathcote et al., 2014; Smith et al., 2014), others have emphasised the importance of understanding the sources of across-trial variability in model parameters (Evans et al., 2020; Jones & Dzhafarov, 2014a). Some researchers have also opted not to include variability parameters in their model and have focused on the most psychologically interpretable parameters such as drift rate and boundary separation (van Ravenzwaaij et al., 2017; Wagenmakers et al., 2007). Others have estimated single-trial drift rates using measures based on stimulus properties (Balsdon & Philiastides, 2024) or neuroimaging data (Turner et al., 2015).

One way to address the ad-hoc criticism is to implement sources of drift rate variability in the model to replace random variability represented by the *η* parameter. In practice, these sources could be exogenous, such as stimulus characteristics and psychometric transformations of how these characteristics affect performance. Sources could also be endogenous, which can be approximated using covert measures of attention or encoding strength on each trial. It is important to note, however, that the random variability can never be fully replaced in practice because drift rate is a latent construct that can never be estimated perfectly on trial level (but see fruitful attempts, Frank et al., 2015; Ghaderi-Kangavari et al., 2023; Holmes et al., 2020; Nunez et al., 2017; Ratcliff et al., 2016).

If random across-trial drift rate variability represents the true variations in the underlying quality of decision evidence, we propose that *η* estimates should decrease and approach zero when endogenous and exogenous sources of drift rate are included in model fitting. Here, we broadly define these sources as ‘systematic variability’ including constructs that are theorised to influence the drift rate, or variables (both behavioural and neural) that differentiate behaviour in ways that can be explained by changes in drift rate. This contrasts with the ‘random variability’ which refers to the residual variability not accounted for by any identified sources of drift rate and is captured by the *η* estimates. Notably, our definitions of systematic and random variability differ from those in previous studies (Evans et al., 2020; Ratcliff et al., 2018). While prior work often defined these terms based on whether variability could be attributed to a specific effect, we define them based on whether they can be practically approximated in the modelling process.

### The Current Study

In the current study, we approximated endogenous and exogenous variables to inform drift rate on each trial; we expected this systematic source of drift rate variability to replace the random drift rate variability—evidence that would suggest that the variability assumption meaningfully accounted for patterns in the data (e.g., slow errors). We attempted to maximise the amount of systematic variability to be incorporated by using a large electroencephalographic (EEG) dataset with ample experimental manipulation and adopt machine-learning to extract information from neural data. It is important to note here that we did not aim to investigate what exact cognitive/neural processes contributes to drift rate or causes slow error, but rather simply to replace the previously-assumed purely random drift rate with trial-level information.

We analysed a large dataset comprising a subset of the Penn Electrophysiology of Encoding and Retrieval Study (PEERS) dataset (Kahana et al., 2023). This includes rich behavioural and electroencephalographic (EEG) data collected during recognition memory tasks using word stimuli. We analysed data from 132 participants with 20 sessions of data collected per participant, with a total of 6,400 trials per person (3,654 on average after EEG data processing). A large dataset with many trials provides better estimates of drift rate variability in DDM (Boehm et al., 2018). There are also a range of manipulations that substantially influence underlying memory strength (i.e., drift rate) such as word frequency, time lag between study and test, and prior recall. Notably, the recall task—where participants tried to recall whatever they remembered from the study list—was administered prior to the recognition task and was understood as a factor that influences memory strength of recalled items.

We approximated endogenous drift rate variability using the EEG signals in the dataset. To maximumly extract information from the EEG data that was related to drift rate, we adopted a machine-learning approach previously used to generate single-trial indices of endogenous drift rates (Philiastides et al., 2006; Ratcliff et al., 2016a). The machine-learning algorithm (logistic regression) first learns the optimal weights for EEG channels that maximise the contrast between two experimental conditions. The optimal weights are then multiplied with the scalp EEG signals at each channel to get a single numerical index which indicates how likely the distributed pattern of signals reflects one condition over the other. Ratcliff et al. (2016a, b) applied this approach in a recognition memory task with EEG recordings, computing EEG measures for each trial that represented either old or new items. They then used these neural indices as regressors to predict drift rate on a trial-by-trial basis using a linear function. Their analysis demonstrated that these indices could explain both behavioural performance and the estimated mean drift rate when the indices were grouped into bins.

Ratcliff et al.’s results also showed a reduction in random across-trial drift rate variability when a single-trial regressor was included (Experiment 2), compared to the standard DDM model. Similarly, Osth et al. (2020) also linked drift rate to averaged similarity of a test item against studied items in a recognition memory task. While the included systematic variability was relatively small, they did not observe reductions in the random variability parameter *η*. These results suggest that endogenous variability may be more strongly represented in the *η* parameter than exogenous variability. Our study allows for a direct investigation of both sources of variability.

In the current study, by linking drift rate on each trial with exogenous and endogenous factors, we expected random across-trial drift rate variability to decrease after incorporating systematic factors, and we aimed to quantify the overall joint variability as we progressively included more trial-level information. We adhered to previous guidelines for estimating the *η* parameter by using a rich dataset with large numbers of trials per participant, using hierarchical Bayesian methods to estimate parameters, and examining parameter recovery (Boehm et al., 2018). To first demonstrate whether the overall joint variability can be partitioned between random and systematic variabilities based on approximated drift rate on each trial, we simulated data with trial-level drift rates derived solely from systematic regressors without random variability and re-fitted the model to simulated data. Additionally, since the regressors are expected to be noisy representations of the underlying drift rate due to measurement errors (especially for endogenous factors), we further tested this approach by adding noise to the data-generating regressors. Finally, we fitted and compared variability estimates from four versions of the model using experimental data: (1) the DDM with only random across-trial drift rate variability, (2) a model with additional exogenous factors explaining trial-by-trial drift rates, (3) a model with only endogenous factors, and (4) a model incorporating all systematic factors. To foreshadow our findings, drift rate variability could be well partitioned into random and systematic components in our simulations even with varying levels of noise added to the regressors. However, the same pattern was not observed when models were fit to experimental data despite the observation that a large amount of systematic variability was accounted for by exogenous and endogenous variables. These findings indicate that other mechanisms apart from drift rate variability are responsible for producing slow errors in this data.

## Method

### Participants

We used an online archival dataset described in Weidemann and Kahana (2019) and used in Sun et al. (2024), which includes data from 132 participants aged 17 to 30 (mean age = 22.1, SD = 0.3). This dataset is part of the Penn Electrophysiology of Encoding and Retrieval Study (PEERS), specifically experiments 1, 2, and 3 (Kahana et al., 2023). Each participant completed recognition tasks over 20 testing sessions during which EEG data was recorded (for more details, see Weidemann & Kahana, 2016). Data collection was approved by the Institutional Review Board of the University of Pennsylvania. Overall, the participants had high performance with a 91.1% hit rate on average (range = 76.5–98.7%, SD = 4.5%) and a false alarm rate of 14.2% (range = 1.7–45.2%, SD = 10.4%). Notably, in this dataset there was slow error pattern observed for targets, with hit responses on average being 253 ms faster than misses, *t*(131) = 37.42, *p* <.001. For lures, we did not observe statistically significant differences in RTs for correct rejections compared to false alarms, *t*(131) = 0.65, *p* =.51.

### Paradigm

**Fig. 2:**
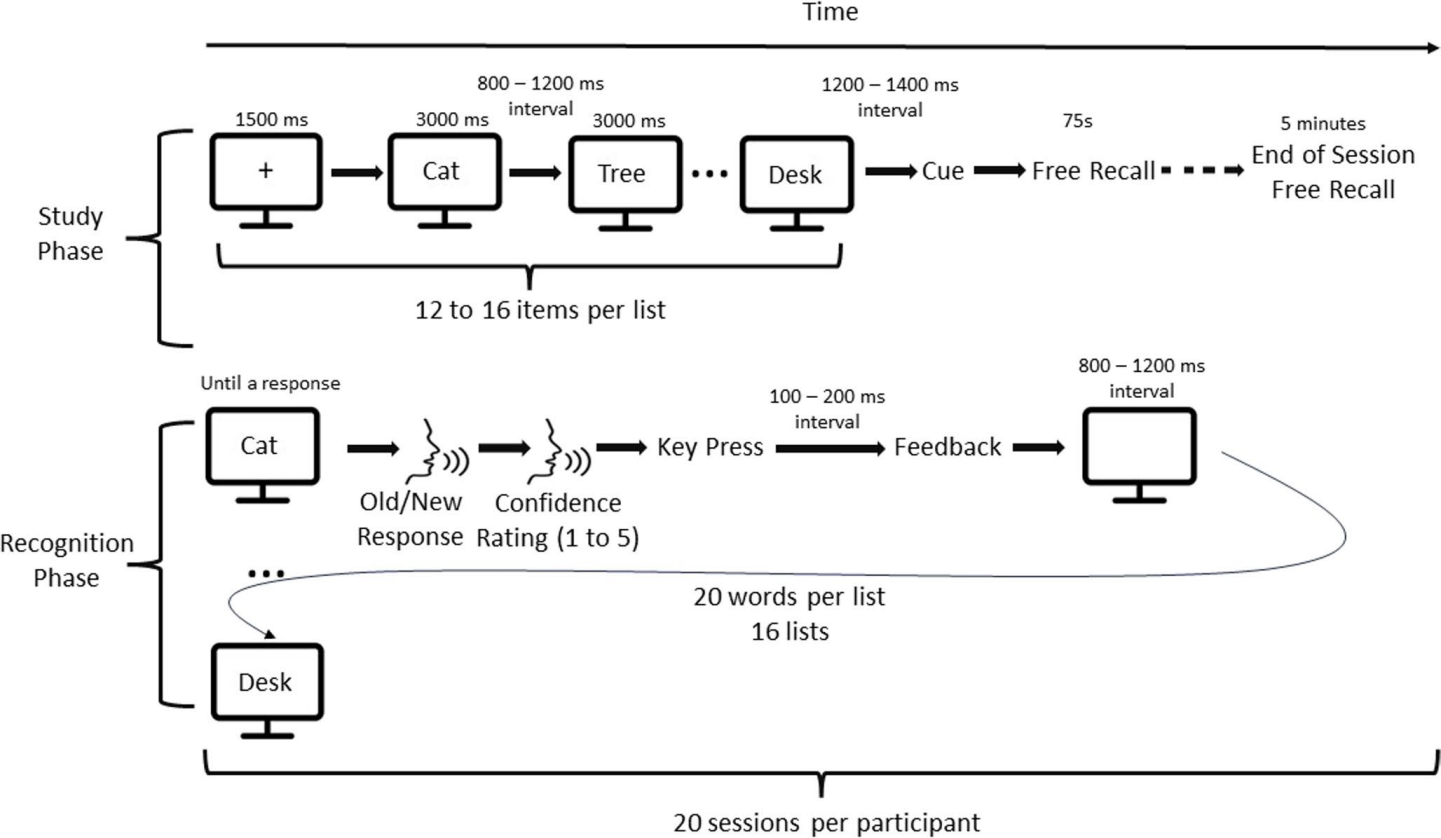
Experiment structure for the dataset described in Weidemann and Kahana (2019)

Throughout the three experiments in the PEERS dataset, different recall and encoding tasks were administered across multiple sessions for each participant (Fig. 2). For detailed descriptions see Kahana et al. (2023). We are only analysing the data related to the recognition task and we will describe only the tasks relevant to our current analyses.

During each session, participants were instructed to study 16 lists, each containing either 12 or 16 words. Each word was displayed for 3 s, with an interstimulus interval between 0.8 and 1.2 s. During the study phase, participants were asked to make size judgments (“Will this item fit into a shoebox?“) or animacy judgments (“Does this word refer to something living or non-living?“) for each word. After studying each list, participants completed an immediate recall task, where they had 75 s to freely recall the items from the list. In half of the randomly selected sessions for each participant, an additional final free recall task was introduced, giving participants 5 min to recall any items they had studied during that session.

After the study and immediate recall tasks, participants completed a recognition memory task. This task involved 320 probe items, with the percentage of targets varying across sessions (80%, 75%, 62.5%, or 50%), while the remainder were lures. Participants verbally responded “Pess” for “yes” and “Po” for “no” to indicate whether they had seen the item in the study lists. They then provided a verbal confidence rating from 1 to 5, indicating how confident they were in their old/new decision for each trial. Feedback on the recognition judgment was given after a 100–200 ms delay following the confidence report, and the next probe item appeared after an interval of 800–1200 ms, which varied between trials.

### Stimuli

The stimuli consisted of 1,638 words used in the PEERS experiments (accessible at https://memory.psych.upenn.edu/Word_Pools). Word lengths varied from 2 to 12 letters (median = 6), and word frequencies, based on the SUBTLEXUS database (Brysbaert & New, 2009), ranged from 0.06 to 5,247 occurrences per million (median = 7.5). For the encoding task, all words were chosen for their clear relevance to both animacy and size judgments.

### EEG Data Acquisition

High-density EEG data were collected using a 129-channel EGI system with a sampling rate of 500 Hz. Channel Cz served as the reference during recording, and all data were re-referenced to an average reference offline. A high-pass filter of 0.1 Hz was applied during recording. The data discussed here were taken from the recognition memory task phase of each session.

### EEG Data Processing

The archival dataset includes 103 channels in total. The twenty-six electrodes that were placed on the face were excluded. In the archival dataset, trials with RTs faster than 300 ms and slower than 3,000 ms were excluded (as done in Weidemann & Kahana, 2019).

We conducted additional data processing using EEGLAB (v 2021.1 Delorme & Makeig, 2004) running in MATLAB (The Mathworks). The pre-processing pipeline was the same as in Sun et al. (2024). Data were processed separately for each session. Epochs were truncated to a time window of −1,000 ms to 2,200 ms relative to target/lure onset. Trials with RTs longer than 2 s were excluded. The data were then low-pass filtered at 30 Hz (EEGLAB Basic Finite Impulse Response Filter New, zero-phase, −6 dB cutoff frequency 33.75 Hz, transition bandwidth 7.5 Hz) and re-referenced to linked mastoids. Channels were flagged as excessively noisy if their amplitudes exceeded ± 500 µV for more than 20% of trials in one session or deviated significantly from the overall channel distribution (> 5 standard deviations). These channels were excluded from further analysis and later interpolated.

Trials with EEG amplitudes exceeding ± 500 µV were rejected. Independent Component Analysis (ICA) was then performed for each session (RunICA extended algorithm, Jung et al., 2000). Components linked to ocular artifacts were identified and removed using ICLabel (Pion-Tonachini et al., 2019). Components classified as eye-related artifacts (with over 85% probability) were subtracted. Excessively noisy channels were interpolated using spherical spline interpolation. The data were baseline-corrected using a 200 ms window before stimulus onset, and trials with amplitudes exceeding ± 200 µV were excluded.

Finally, EEG data were aggregated across all sessions for each participant. The stimulus-locked segments used in the analyses spanned from − 1,000 to 2,200 ms relative to stimulus onset. The code for data processing and analysis, along with the processed data, is available at https://osf.io/r69ws.

### Endogenous Factors Estimated Using EEG Data

To generate a measure of EEG that approximates the endogenous variability of drift rate, we adopted a single-trial regressor approach (Philiastides et al., 2006; Ratcliff et al., 2016a, b). For each trial, regressors were computed based on averaged EEG amplitudes over 100-ms time windows. We selected three time windows spanning 500 to 800 ms in the stimulus-locked epochs to generate three regressors. The time windows were consistent with the previous findings of event-related potentials (ERPs) in recognition memory tasks that are associated with differentiation of correct rejection of lures and correct recognition of targets (Rugg and Curran, 2007). The selection of time windows relative to the memory probe onset was also consistent with findings of Ratcliff et al. (2016a, b) that showed a strong association between single-trial drift rates and EEG regressors.

To compute the single-trial regressors, we followed Ratcliff et al. (2016a, b) to contrast old and new items for optimising the channel weights. This contrast constitutes an item-based approach capturing the neural patterns that discriminates old and new items. A higher regressor value indicates a more ‘old-like’ pattern of EEG data. This is assumed to be associated with greater likelihood of making an ‘old’ response during the recognition decision, therefore related to a higher drift rate.

The single trial regressor y at a time window (T) is calculated as:

1 $$\:\begin{array}{c}Y\left(T\right)=w^Tx\left(T\right)\end{array}$$

where w is the vector of weights for all the EEG channels included in the analysis and x is the vector of channel values averaged in time window T. We assumed the old and new conditions are distributed as a logistic function, and the likelihood for each condition given the averaged EEG values is calculated as:

2 $$\:\begin{array}{c}p\left(c=old\:\right|\:x)=\frac1{1+e^{-w^Tx\left(old\right)}}\end{array}$$

3 $$\:\begin{array}{c}p\left(c=new\:\right|\:x)=1\:-\frac1{1+e^{-w^Tx\left(new\right)}}\end{array}$$

The optimal weights are obtained by minimizing the negative sum likelihood of the data. This was done using a differential evolution method based on the algorithm of Storn and Price (1997). Differential evolution is a simple yet robust optimizing method that explores large parameter spaces which is useful as we are estimating a vector of 103 channel weights. Therefore, this method helps to avoid the optimization being stuck at a local minimum. We calculated the receiver operator characteristics and the area under the curve to measure classification accuracy. For the 100-ms time windows from 500 to 800 ms, the mean classification accuracies across participants were 61.3% (SD = 4.1%), 66.7% (SD = 5.0%), and 69.4% (SD = 4.5%), suggesting later time windows were more strongly associated with behavioural performance. This was also consistent with classifier performances observed in previous studies of recognition memory tasks with EEG data (Ratcliff et al., 2016a, b; Weidemann & Kahana, 2019). We further performed a 5-fold validation procedure. For the three window, the averaged classification accuracies over the 5 validation samples were 60.58%, 62.84%, and 64.26%, respectively. Therefore, we suggest that the current machine-learning model could reliably differentiate between old and new words.

We further included the EEG alpha band power prior to the stimulus onset as another endogenous factor contributing to drift rate. This pre-stimulus alpha has been broadly suggested to index attentional state (May et al., 2012; Slagter et al., 2016), such as the level of vigilance (Minkwitz et al., 2011). While it is generally expected that task performance will be better when participants are attentive, within DDM, attentional changes/mechanisms have been implemented to primarily affect the evidence accumulation process (Nunez et al., 2017; Tavares et al., 2017). Hence, we calculated the alpha band power over a one-second time window prior to the stimulus onset at electrode Oz.

### Exogenous Factors

As mentioned above, we selected word frequency, prior recall, and study-test time lag as exogenous factors. First, we log-transformed the SUBTLEXUS word frequency to reduce the range of this variable (mean = 2.01, standard deviation = 1.53). Studies have indicated that memory strength for words varies according to their natural frequency in language. For example, high-frequency target words are less likely to be correctly recognized than low-frequency words, while high-frequency lures are more likely to be falsely recognized as old (Glanzer & Adams, 1985). Recognition memory task performance related to word frequency for the same dataset has been also analysed in a previous study (Lohnas & Kahana, 2013), which showed higher hit rates and lower false alarm rate for lower word frequency items and the relationship was reasonably monotonic.

Second, we estimated for each test probe the time lag between when it was encoded during the study phase and tested in recognition memory task, (mean = 21.5 min, SD = 7.9 min). Ample evidence suggested that memory performance for items decreases over time following a power-law function after encoding (Wixted & Ebbesen, 1991). The calculation was based on the precise time of each test probe presentation relative to the start of the testing phase, and the estimated study-test time lag for each item based on its encoding list number and position within the list. This was because we did not have access to the study phase data and the precise timing of each encoding phase stimulus presentation.

Lastly, the immediate free recall was coded as a binary variable that indicates either a success or failure of recall for a studied item. Prior recall of an item has been shown to improve subsequent recognition (Chan & McDermott, 2007), which could indicate a greater memory strength of the recalled item.

### DDM Parameterisation

All the versions of DDM we fit were extensions to the ‘standard model’ which included the following parameters: starting point *z*, boundary separation *a*, non-decision time *T*_*er*_, and the across-trial variability parameters for non-decision time *S*_*t*_, drift rate for targets *V*_T_, and lures *V*_L_. Separate drift rate variability parameters for targets and lures (*η*_T_ and *η*_L_) were included as prior work has estimated targets to have larger drift rate variability than lures (Osth et al., 2017; Starns, 2014; Starns & Ratcliff, 2014). We did not include across-trial variability in the starting point as the inclusion of this parameter was not found to contribute to the estimation of drift rate variability, and such parameterisation is generally recommended for recognition memory tasks (Osth et al., 2017). This is because fast errors are not commonly observed in recognition memory tasks. We did not let *z* and *a* vary across targets and lures as we assumed decision bias and caution would be consistent across randomly ordered target and lure presentations during the test phase. We included non-decision time variability as it allows the model to capture changes in the leading edge of the RT distribution in addition to changes in drift rate (Ratcliff et al., 2004; Ratcliff & Tuerlinckx, 2002). The leading edge is commonly defined as the fastest 0.1 quantile of the RT distribution. With the inclusion of non-decision time variability, fast responses are more likely in trials with a combination of high drift rates and short non-decision times. This leads to a steeper rise in the leading edge of the predicted RT distribution.

To include the exogenous and/or endogenous variables of drift rate in trial-by-trial model fitting, we used a regression approach that regresses approximated systematic factors to drift rate. For a given trial, the drift rate *V*_ij_ for trial *i* in condition *j* was regressed onto different functions describing how each systematic factor was related to the drift rate as follows:

4 $$\:\begin{array}{c}v_{ij}=\:v_{j\:}+f_1\left(x_{1ij}\right)+\dots\:+\:f_n\left(x_{nij}\right)\end{array}$$

5 $$\:\begin{array}{c}f\left(x_{ij}\right)={\beta\:}_jx_{ij}\end{array}$$

where *V*_j_ is the intercept of the regression model for condition *j*, *x* is the included variable values assuming a functional relationship with drift rate denoted by *f*. For most of the factors (word frequency, prior recall and endogenous factors), a linear function was used, and beta parameters are the regression coefficients estimated for each variable for condition *j*. For the word frequency variable, beta parameters were separately estimated for targets and lures given that word frequency effects differ between them. This was also done for all the endogenous variables to inform drift rates from neural processes associated with targets and lures. For study-test lag and recall, parameters were only estimated for targets. Prior recall was modelled as a linear function for a binary variable. Study-test lag (t) was modelled using a power-law forgetting function as:

6 $$\:\begin{array}{c}f\left(x_{ij}\right)={{\alpha\:}_jt_{ij}}^{-k_j}\end{array}$$

where *α* and *k* were parameters that constrain the shape of the forgetting curve. These parameters were separately estimated for recalled and unrecalled target words. This is because items that were recalled prior to the recognition memory task were associated with a greater memory strength from a successful retrieval which would likely reflect different functions when placed on the same time scale.

Four versions of the DDM were constructed: a model with only random drift rate variability (standard model), and three models with random drift rate variability and additional trial-level regressors: a model with only exogenous factors (exogenous model), a model with only endogenous regressors (endogenous model), and a model with all regressors (complete model, see Table 1 for parameters in each model).

**Table 1:** Parameters for all models fitted to experimental data

Models	Parameters
Standard model	*z*,* a*,* T*_*er*_, *S*_*t*_, *V*_T_, *V*_L_, *η*_T_, *η*_L_
Exogenous model	*z*,* a*,* T*_*er*_, *S*_*t*_, *V*_T_, *V*_*L*_, *η*_T_, *η*_*L*_, *β*_wfT_, *β*_wfL_, *β*_recall_, *α*_recalled_, *k*_recalled_, *α*_unrecalled_, *k*_unrecalled_
Endogenous model	*z*,* a*,* T*_*er*_, *S*_*t*_, *V*_T_, *V*_L_, *η*_T_, *η*_L_, *β*_EEGT*1*_, *β*_EEGT2_, *β*_EEGT3_, *β*_EEGTA,_ *β*_EEGL1_, *β*_EEGL2_, *β*_EEGL3,_ *β*_EEGLA_
Complete model	*z*,* a*,* T*_*er*_, *S*_*t*_, *V*_T_, *V*_L_, *η*_T_, *η*_*L*_, *β*_wfT_, *β*_wfL_, *β*_recall_, *α*_recalled_, *k*_recalled_, *α*_unrecalled_, *k*_unrecalled_, *β*_EEGT*1*_, *β*_EEGT2_, *β*_EEGT3_, *β*_EEGTA,_ *β*_EEGL1_, *β*_EEGL2_, *β*_EEGL3,_ *β*_EEGLA_

*Subscript wf*  word frequency, *T* target, *L* lure, *A* Alpha band. *Subscripts EEG1 – EEG3* refer to the three 100-ms time windows from 500 to 800 ms after the memory probe onset

 The amount of systematic variability was then calculated by applying the regression equation with trial-level regressors to the data. For each participant on each trial, we calculated the drift rate estimate according to equation 4. The systematic drift rate variability was estimated by taking the standard deviation of the trial-level drift rates for each participant. This was done repeatedly for each posterior sample. Therefore, the overall joint drift rate variability was calculated as:

7 $$\:\begin{array}{c}Joint\:variability=\sqrt{\:systematic\;variability^2+\:{\eta\:}^2}\end{array}$$

### Hierarchical Bayesian Modelling

We estimated the parameters from the models using a hierarchical Bayesian approach that simultaneously estimates individual and group-level parameters. This approach allows uncertainty in parameters to be estimated from posterior samples and provides better estimates of individual level parameters constrained from group level parameters through ‘shrinkage’. We produced the posterior distribution using the differential evolution Markov Chain Monte Carlo algorithm (DE-MCMC, Turner et al., 2013) that simultaneously generates vectors of parameters across multiple MCMC chains. For each sampling step in each chain, the DE-MCMC algorithm produces new parameter proposals based on information from other chains, which has been suggested to be robust to parameter correlations. Details regarding the prior distributions of parameters are included in Appendix (A) The detailed model fitting procedure is described in Appendix (B) Because drift rates vary across trials, we estimated the DDM likelihood for each trial and summed the log-likelihood for each participant; this was done by calculating density functions implemented in fast-dm-30 (Voss et al., 2015).

We inspected the posterior sample convergence by examining the Gelman-Rubin (GR) convergence statistics that calculate the ratio between inter- and intra-chain variance. Convergence is determined by whether the GR statistic falls below 1.1 along visual inspections of trace plots of posterior chains. The comparison of models was judged based on the Widely Applicable Information Criterion (WAIC, Watanabe & Opper, 2010), which balances between the model goodness-of-fit and model complexity. WAIC is preferred over other information criteria (e.g., Deviance Information Criterion) because WAIC integrates uncertainties over the posterior parameter estimates rather than relying on point estimates, and approximates leave-one-out cross-validation, a standard method for model selection. Models with a smaller WAIC value represent a better goodness-of-fit with consideration of model simplicity.

### Recovering Drift Rate Variability with Simulations

We also tested the feasibility of the regression approach to account for drift rate variability with simulations. We fitted a DDM to the experimental data where drift rate was determined only by systematic variables without random across-trial drift rate variability, and we then simulated data using this model. That means, for the simulated data, we can access the ground truth drift rate for each trial and the distribution across trials in the simulated data. Additionally, any slow error pattern in the simulated data would be solely due to the drift rate distribution across trials. We then fitted the models with either random variability, exogenous variability and both exogenous and endogenous variability to this simulated data, and examined whether the drift rate variability can be reliably recovered and partitioned into random and systematic components of drift rate.

### Selection of Regressors for Simulation

As we aim to recover the variability estimates based on the DDM that assumes a normal distribution of drift rate, we also observed that the systematic variability functions can produce data-generating drift rate distributions that largely deviate from a normal distribution. To investigate potential biases in this simulation-based parameter recovery and select appropriate functions for simulations, we tested the effect of drift rate distribution skewness on the recovery of the *η* parameter. We examined the model recovery of the drift rate variability parameter for parent distributions that were normal distributions with same mean and standard deviation but with either positive (skewness = 0.78, kurtosis = 0.63), negative (skewness = 0.99, kurtosis = 0.87) or no skewness.

Given the extensive model fitting required for this analysis, the models were fitted using maximum likelihood method with a differential evolution algorithm implemented in Python (Scipy package, strategy = ‘best2bin’, recombination rate = 0.9). We generated 20,000 trials with equal numbers of targets and lures based on parameters estimated from hierarchical Bayesian models. For each skewness condition, we fitted 18 models with different bias, drift rate and variability conditions (recovered drift rate parameters are displayed in Appendix C, Table 3). This allowed us to systematically evaluate the effect of skewness on parameter recovery.

**Fig. 3:**
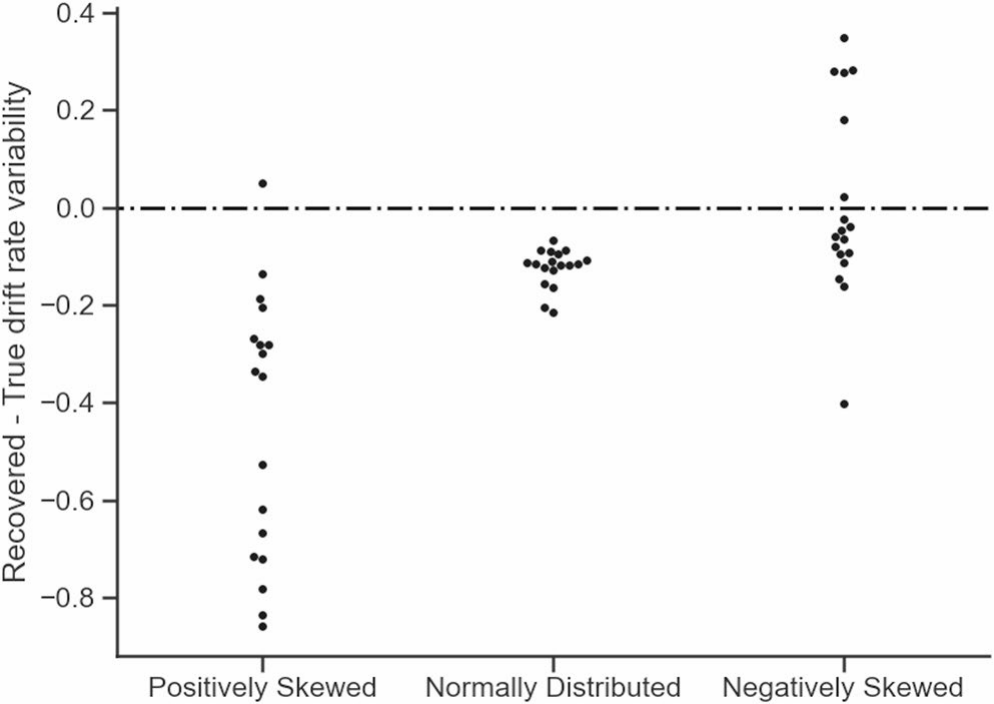
Recovered across-trial drift rate variability parameters across different data-generating conditions. The dots represent the difference between the recovered and the data-generating parameter

We found that, for both low and high drift rate conditions, the drift rate variability parameter tends to be under-estimated for positively skewed parent drift rate distributions (Fig. 3). For this reason, we excluded the time lag function that produces skewed drift rate distributions (skewness = 0.86, kurtosis = 0.73) and the recall function that produces a bimodal distribution. Therefore, we included the word frequency function and 5 EEG measures (from 300 to 800 ms relative to stimulus onset) for simulations based on only systematic variations.

The effect of skewness in the underlying drift rate distribution on the estimation of *η* in the standard DDM has not been systematically investigated before. There have been investigations of other distributions (Ratcliff, 2013) but these were symmetrical. As the true shape of underlying drift rate distribution in the data is unknown, we tested whether systematic variability from experimental data could better inform the model under a skewed distribution assumption compared to the standard normal distribution. Previous studies of memory have adopted a positively skewed distribution assumption for memory strength (Shiffrin & Steyvers, 1997a; Shimamura & Wickens, 2009) indicating that relatively few items are well remembered. Applying this assumption in the recognition memory task, we have additionally tested a shifted log-normal distribution assumption with a positive skewness to replace the normal distribution for the *η* parameter (see supplementary materials for more details). The shifted log-normal distribution is the exponential of normal distribution with an additional parameter to horizontally shift the distribution, allowing for negative values for drift rates. To summarise the results, we did not find a substantial amount of skewness estimated from model fitting.

## Results

To preview our results, we first validate the use of the single-trial regressor approach for explaining drift rate variability by presenting parameter recovery analyses based on simulated data. We then explore, through simulations, the impact on the drift rate variability estimates when the random noise is added to the regressors. Following this, we evaluate the fits of the models to the experimental data, assessing how well they capture observed trends. Finally, we examine the estimates of both systematic and random drift rate variability derived from these models.

### Parameter Recovery from Simulations

**Fig. 4:**
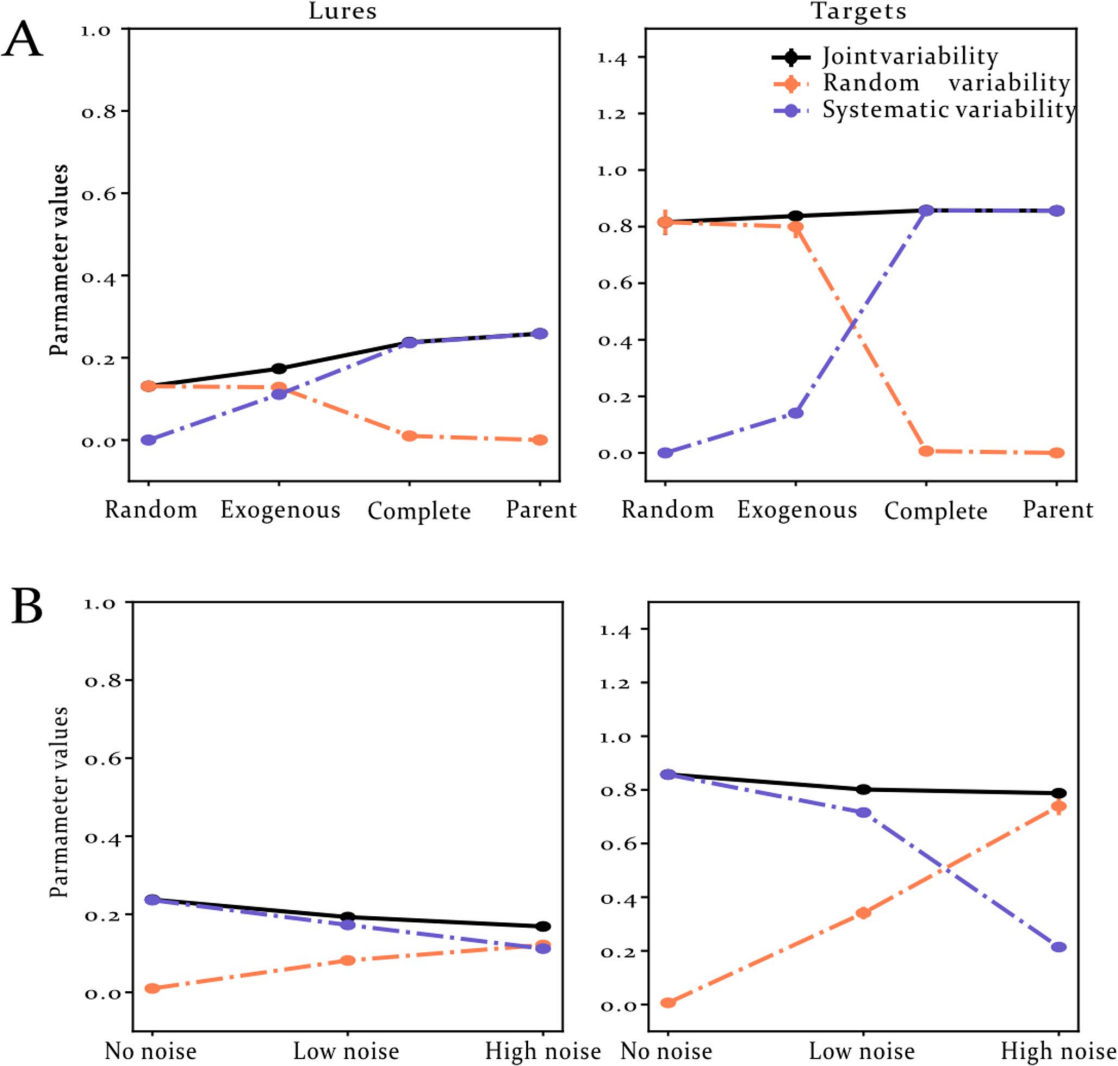
Parameter recovery of *η* parameters based on simulated data that were generated from (**A**) regressors estimated from data and (**B**) same regressors with varying degree of added Gaussian noise. The error bar represents the 95% highest posterior density interval. For subplot A, Random = standard DDM, Exogenous = DDM with only exogenous regressors, Complete = DDM with all regressors. Parent = data-generating model. For subplot B, the recovery was based on the complete model and data generated from the parent model, but with added noises to the endogenous regressors

We examined whether the underlying drift rate distribution can be reliably recovered when the data-generating drift rate distribution was systematic and caused slow errors. This was an important validation test for the current methodology to explain drift rate variability, and the results would also provide us confidence in the interpretation of model fits to real data. We inspected how random, systematic, and joint variability changes as more systematic variability was included. The joint variability was calculated as square root of the sum of the random and systematic variances. We observed that the overall drift rate variability for targets could be reliably recovered by a combination of random and systematic drift rate components (depicted in Fig. 4A). Specifically, we observed that the *η* parameter dropped close to zero when all the data-generating regressors were included in the recovery. For lures, while the overall variability could be recovered when all the data-generating factors were included, it was underestimated when drift rate was partially or not informed (i.e., random and exogenous models). This could be due to a low variability presented in lures which leads to small behavioural effects that are difficult to be captured by the *η* parameter in the model. Overall, this indicates that our approach to use systematic factors to explain random drift rate variability is feasible when the underlying across-trial drift rates are normally distributed and produce a clear slow error pattern.

In the current regressor approach, we did not include an error term in the linear regression to account for measurement errors in the regressors. This is a simplifying assumption, implying that variations in the regressors fully represent true variations in the drift rate, which is not the case. To test the effect of additional measurement noise in the regressors, we simulated data with the original regressors serving as the ground truth and tried to recover the drift rate variability with the noisy versions of the regressors. Specifically, we added different levels of unbiased Gaussian noise to each endogenous regressor, with a mean of zero and a standard deviation of either 0.5 (low noise) or 3 (high noise). In comparison, the endogenous regressors had means of zero and standard deviations of 1. When comparing the recovery of the drift rate from noisy regressors to that of the original regressors, we observed a decrease in the systematic variability incorporated into the model (Fig. 4B). However, the overall variability remained relatively constant, with random variability compensating for the reduction in systematic factors. This suggests that the current regression approach is capable of leveraging noise in the regressors, and reducing the influence of systematic factors if they are noisy, without affecting the overall estimation of drift rate variability.

While the slow errors in the current simulations were solely caused by across-trial drift rate variability assumption, as discussed, other sources of slow errors have been proposed. To understand the effect of these unknown sources if they are present, we approximated this effect in the simulations by artificially further slowing the error responses (Supplementary Material). We observed inflated estimates of overall drift rate variability across models, consistent with previous findings that suggested *η* parameter estimation was driven by slow errors in the data (Yap et al., 2012).

### Models Fitted to Experimental Data

We examined both quantitative fits based on model selection criteria as well as qualitative fits indicated by the match between data and model predictions. Across the standard, exogenous, endogenous and complete models, the models that included more regressors were preferred based on the WAIC values (Table 2) with very large WAIC differences across models. As WAIC is calculated on a log scale, a difference of 10 was considered to be large. Regardless of the sources of across-trial drift rate variability, all models were able to capture the slow errors in the data due to the inclusion of the variability assumption (see Supplementary Material). Group-level posterior parameter estimates for each model were in Appendix D (Table 4).

**Table 2:** WAIC scores for each model

Models	Standard Model	Exogenous Model	Endogenous Model	Complete model
WAIC	114,803	88,082	58,828	37,537

**Fig. 5:**
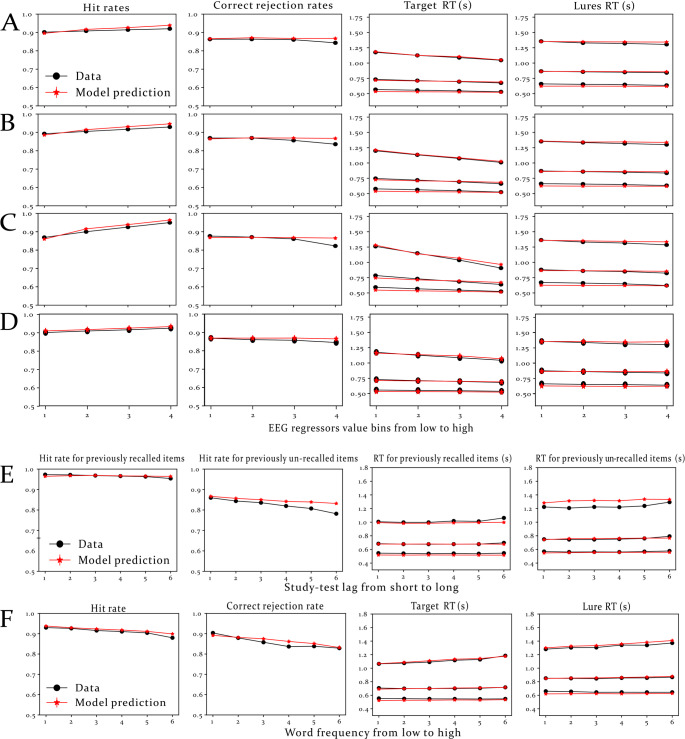
Model predictions against regressors binned by values. Model predictions of binned EEG regressors values extracted based on (**A**) 500 to 600 ms time window, (**B**) 600 to 700 ms time window, (**C**) 700 to 800 ms time window after stimulus onset for targets and lures, and (**D**) pre-stimulus EEG alpha band power for targets and lures. (**E**) Model predictions of targets varying between study-time lag and whether the item was previous recalled. (**F**) Model predictions of binned log word frequency values for targets and lures

Further, we checked the model predictions with respect to each regressor we included in the model. Model predictions from the complete model binned by regressor values are plotted in Fig. 5. This model demonstrates the amount of overall variability that could be estimated from both exogenous and endogenous variables. Overall, we observed that the complete model was able to capture the changes in accuracies and RTs associated with each manipulation; the model predictions closely matched the trends in the data on the group level when sorted by values of each regressor. For exogenous factors, the model was able to predict almost perfect recognition across participants if an item was previously recalled prior to the recognition task compared to un-recalled items and recognition accuracy decreased with longer lags between study and test (Fig. 5D). It was also noted the recognition accuracy for unrecalled items decreased faster with study-test lag given that recalled items were recently retrieved from memory. Second, the model was able to capture the effects of word frequency for both targets and lures, where higher frequency words were associated with lower accuracy and slower RTs for the slowest quantile (Fig. 5E), which was consistent with previous observations from analyses of the same dataset (Weidemann & Kahana, 2016).

For the endogenous factors, we observed that more variation in performance was accounted for by EEG measures from later time windows relative to memory probe onset (Fig. 5A - C), in which greater regressor values were associated with more accurate decisions and faster RTs. This was consistent with our observations of higher classification accuracies from machine learning for later time windows. These trends were also captured by the model predictions. Our results were consistent with previous studies using the same method in which large performance differences were observed across different regressor values (Philiastides et al., 2006; Ratcliff et al., 2016a). Overall, these observations also suggest that our implementation of single-trial regressors was able to help the model systematically explain variation in performance.

### Drift Rate Variabilities Estimated from Experimental Data

**Fig. 6:**
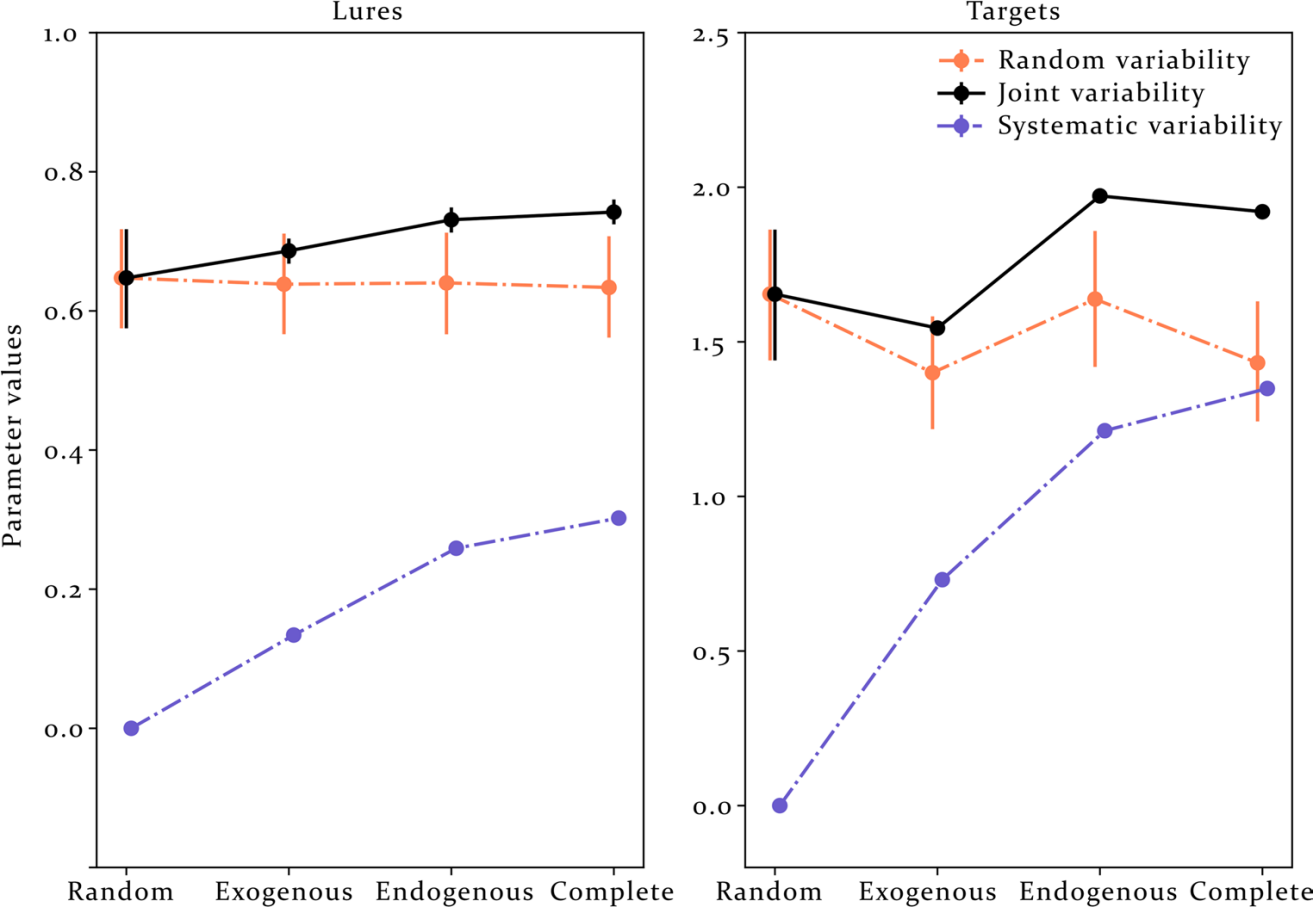
Model-estimated *η* parameters based on experimental data. The joint variability is calculated as square root of the sum of the random and systematic variances. The error bar represents the 95% highest posterior density interval. Random = standard DDM, Exogenous = DDM with only exogenous regressors, Endogenous = DDM with only endogenous regressors, Complete = DDM with all regressors

The drift rate variability estimates from fitting the models to the experimental data showed that a large amount of systematic variability was accounted for by the models (Fig. 6). Given that we included more factors for targets than lures, it is perhaps not surprising that we observed more systematic variability for targets. Compared to the random drift rate variability estimated by the standard DDM without systematic drift rate factors (based on the mean of the posterior distribution), the proportional amount of systematic variability increased from the exogenous to the endogenous and complete model for both lures (20%, 40% and 48%) and targets (45%, 72% and 81%). This suggests that the models could incorporate a large amount of total variability estimated by the standard model. Further, the amount of variability included in the current study also largely exceeds a previous attempt to include exogenous variability in a recognition memory task (Osth et al., 2020). Therefore, we suggest that current implementations accounted for a substantial amount of variability in drift rate in the dataset.

However, the estimation of the random variability parameter did not show a corresponding decrease similar to that which was observed in our simulations (Fig. 4). The inclusion of systematic variations did not substantially change the estimations of *η* parameters across targets and lures (Fig. 6). Specifically, the *η* estimates for lures stayed relatively constant across models tested. For targets, the reductions in *η* for targets were small when compared to the amount of systematic variability included. We also note the reduction in *η* only occurred for the exogenous and complete model when compared to the standard DDM. Therefore, the overall joint variability estimates did not stay constant as observed in our simulations, but changed with the amount of systematic variability.

For endogenous factors, it is expected the estimated systematic variability contains more noise due to the nature of EEG data. However, it was shown that variability in the endogenous factors accounted for a large amount of variance in accuracy and RTs, and the endogenous model was able to predict this variance (Fig. 4). As our simulations showed lower estimated systematic variability with higher amount of noise in the factors, the fact that a large amount of systematic variability was estimated for targets indicates the endogenous factors are good indicators of trial-level drift rate.

We have additionally considered a possible role of a skewed drift rate distribution in estimating the variability estimates under the normal distribution assumption in DDM. While we have found evidence showing underestimated random drift rate variabilities when the underlying distribution was positively skewed (Fig. 3), we have also tested whether there was a benefit to assume memory strength distribution to be positively skewed in modelling recognition memory (Supplementary Materials). To do so, we have replaced the normal distribution with a shifted log-normal distribution that was naturally positively skewed. However, we found little skewness estimated with parameters of this distribution, therefore little evidence for a positively skewed distribution assumption in the DDM for the data collected during the recognition memory task.

In summary, the results from fitting the models to experimental data deviated from our expectations based on simulations. Specifically, random drift rate variability was only minimally reduced despite the inclusion of a substantial amount of systematic drift rate variability. Moreover, the observed patterns were inconsistent with the effects of noisy regressors demonstrated in our simulations, which indicated a reduced contribution of systematic drift rate variability.

## Discussion

We aimed to investigate the drift rate variability parameter in the DDM as a mechanism for accounting for slow errors. Specifically, if the *η* parameter represents the true variability in the quality of the decision evidence that also causes slow errors, we should expect its estimates to decrease after accounting for systematic variability. We assessed this by using single-trial regressors generated from endogenous and exogenous variables to inform drift rate on each trial. Based on simulations, we first demonstrated the feasibility of this approach through parameter recovery; when drift rate variability is the sole source of slow errors, it can be accounted for by trial-level information. Furthermore, we showed that even when the regressors are noisy representations of the trial-level information, the joint variability can still be effectively partitioned between random and systematic components, albeit with an increase in estimated random variability. However, this result contrasts with what we observed when applying this method to a large experimental dataset. We found little change in random across-trial drift rate variability estimates as more systematic variance was included in the models. Therefore, our findings suggest that random across-trial drift rate variability remains necessary for the model to account for slow errors, even after including systematic variability. We interpret this as evidence for the presence of other potential mechanisms contributing to slow errors in the recognition memory task, which limits the meaningful interpretation of the drift rate variability parameter.

Our findings are consistent with previous studies that suggested the drift rate variability parameter estimation is driven by the slow error pattern (Ratcliff & Tuerlinckx, 2002; Yap et al., 2012). We have additionally demonstrated this property of DDM with simulations (Supplementary Material). This suggests while the *η* parameter is theoretically assumed to represent the underlying distribution of decision evidence, its estimation is practically constrained by slow errors, and its interpretation is therefore limited by whether the distribution of decision evidence is responsible for slow errors. Our simulations of the DDM with only systematic variability have demonstrated this property. In this model, slow errors were solely caused by the implementation of drift rate variability and the recovery of the drift rate variability was accurate. These findings suggest that the random across-trial drift rate variability parameter can be meaningfully interpreted as reflecting true underlying variability if slow errors are caused by the across-trial variability in the drift rate and are present in the data.

However, we did not observe a similar pattern when fitting models that included systematic drift rate information to the experimental data. While the inclusion of regressors accounted for a large amount of systematic drift rate variability and helped the model improve trial-level predictions (shown in Fig. 5), we did not observe a substantial decrease of the random across-trial drift rate variability parameter and a stable estimate of the overall variability. This suggests that a substantial amount of random across-trial variability is still needed to account for the patterns in the data. Notably, our results contrast with the results in Ratcliff et al. (2016a, b) where a large reduction was observed. Different to their methods, we used a large dataset and state of the art model fitting algorithm for posterior parameter estimation across all the models. We argue that the property of DE-MCMC have helped us to explore a wide parameter space and achieved stabilised parameter estimates. Therefore, we suggest other potential sources of slow errors could be present in the data which was accounted by the model with drift rate variability.

As mentioned above, there are other mechanisms implemented in the evidence accumulation models that produce slow error patterns. These include time-variant parameters for the within-trial accumulation process that increase error rates for longer accumulation durations (Asadpour et al., 2024; Hawkins et al., 2015), lateral inhibition across accumulators (Usher & McClelland, 2001; Yi, 2020) and the racing structure for multiple accumulators (Hawkins & Heathcote, 2021; Tillman et al., 2020). These mechanisms share a commonality regarding how they produce slow errors, which necessitates a slow rate of evidence accumulation (e.g., a low drift rate in the error accumulator for multi-accumulator models). The differences across these mechanisms lie in how the errors are generated within a trial. While models like the DDM and racing diffusion models rely on a random diffusion process that probabilistically predicts higher error rates for longer responses, the LCA and time-variant models provide explanations based on specific neural or cognitive processes (i.e., lateral inhibition and urgency). However, formal modelling of the urgency mechanism was shown not to be preferred over the DDM with or without drift rate variability, and that the urgency mechanism could not predict slow errors as well as the version of DDM with drift rate variability (Evans et al., 2017; Smith & Ratcliff, 2022; Winkel et al., 2014).

Lateral inhibition has been supported by observations of inhibitory mechanism in the neural populations in human perception (Meinhardt & Gierer, 2000). Notably, the inclusion of lateral inhibition in the Leaky Competing Accumulator (LCA) model does not inherently cause slow errors; it is suggested that slow errors only occur when lateral inhibition dominates the accumulation process, particularly under conditions of lower evidence levels and leakage (Yi, 2020). In terms of memory processes, previous studies have suggested a plausible inhibition process during retrieval from long-term memory, where the activation of competing memory traces is suppressed (Levy & Anderson, 2002). Therefore, lateral inhibition presents itself as a plausible candidate for explaining slow errors without the need for additional across-trial distributional assumptions. However, testing the LCA model in the current study was not feasible due to its lack of a closed-form likelihood function and recovery issues (Miletić et al., 2017). A recent proposal of using neural networks to study relationships between model parameters and model predictions allowed parameter estimation for models without closed-form likelihood (Radev et al., 2020), which offers opportunities to test novel implementations without analytic solutions. Apart from testing additional mechanisms of slow errors, errors trials could also be systematically related to slower non-decision time; this could be investigated by using other neural signatures to inform trial-level non-decision time parameters (Nunez et al., 2019).

To further scrutinize our results, we also considered potential issues with our current approach. Specifically, by regressing both signal and noise onto the drift rate, it remains unclear how this might affect the estimation of random across-trial drift rate variability. To address this, we adopted a simple approach to test our method. We simulated data from the model without random across-trial drift rate variability, using the ground-truth regressors, and then tested the recovery using the same regressors but with added Gaussian noise. However, we did not observe the same results as when fitting the model to real data. The systematic variability was estimated to be low as the trial-level regressors became less informative, while the overall drift rate variability remained constant. This suggests that our observation—that random variability was not fully accounted for by systematic variability—was unlikely due to this misspecification. Recent studies that used BayesFlow (Radev et al., 2020) for joint modelling behavioural and neural data with DDM offered some flexibility, in which the measurement noise of the neural data was specifically modelled while the ‘true’ trial-level neural index informs DDM parameters on each trial (Ghaderi-Kangavari et al., 2023). However, since research using this method is still in its early stages, there has not yet been an attempt to validate the measurement noise parameter.

In addition to our main findings, we also investigated the effect of skewed distributions on the recovery of random across-trial drift rate variability parameters. While the assumption of a normal distribution was reasonably met for our simulation, we found that the recovery of the *η* parameter could be systematically biased by the skewness of the underlying drift rate distribution. Specifically, the *η* parameter tended to be underestimated for a positively skewed distribution, with stronger biases observed at higher drift rates and variabilities (Appendix C). While previous studies have examined symmetrical distribution assumptions (Ratcliff, 2013), for the first time, we tested an asymmetrical distribution (shifted log-normal distribution) of drift rate in DDM.

Notably, in recognition memory, some models include assumptions relating to a skewed distribution of memory strength. For example, Shimamura and Wickens (2009) adopted an ex-Gaussian SDT that assumes the distribution for item memory is positively skewed. They argued the skewed distribution assumption was the product of the hierarchical structure of the medial temporal lobe and relational binding principles, which suggests that memory strength for better remembered items increases disproportionately. Further, in the Retrieving Effectively from Memory (REM) model (Shiffrin & Steyvers, 1997a), episodic memory is assumed to be stored as vectors of features. The distribution of these features follows a geometric distribution with a positive skew, implying that diagnostic features are rarer than common features. During retrieval for recognition memory, the features of the probe item are compared with memory traces and generates a measure of memory strength, and the distribution of the memory strength was suggested to be positively skewed. Importantly, the implication of a skewed distribution of memory strength in REM was a product of feature matching and global similarity computation instead of a convenient implementation.

In our current observations, it is possible that the failure to account for random variability with systematic variability was due to an underlying skewed distribution of memory, leading to biased *η* parameter estimation. To investigate this, we tested a version of the DDM with a shifted log-normal drift rate distribution. Shifted log-normal distributions exhibit positive skewness, and their logarithmic form follows a normal distribution. However, we did not observe strong skewness based on the distribution parameters. This suggests that the log-normal distribution was approximating a normal distribution. While implementing a skewed distribution assumption in the DDM has not been done before, this preliminary evidence suggests no advantage to such an implementation regarding variability estimation for drift rate. This further supports the idea that our current observations are likely due to other potential sources of slow errors present in the data.

Finally, we want to re-iterate that our findings do not oppose the idea of variable evidence across different trials or decisions. Most if not all the existing models of memory assumes varying degrees of memory strength for each retrieved item, based on memory representations and/or retrieval processes (for a review, Osth & Dennis, 2024; Shiffrin & Steyvers, 1997b). For recognition memory tasks, a greater variability in memory strength for targets compared to lures has been universally found with SDT models and the DDM (Chen et al., 2024; Osth et al., 2017; Starns & Ratcliff, 2014). More recently, researchers have also moved to use realistic representations of the item features in recognition memory tasks to help better calculate trial-level item similarities and derive predictions of memory performance (Cox et al., 2018; Osth et al., 2020; Osth & Zhang, 2024). It would be useful if the variability in memory strength could be better estimated from a process model. However, our findings caution the difficulty in drawing such an inference based on the *η* estimates in standard DDM because the variability assumption might not be the sole cause of slow errors.

## Conclusion

In this study, we showed that the random drift rate variability as implemented in the DDM cannot be adequately accounted for by systematic variability, and that estimation of across-trial drift rate variability can be influenced by slow error patterns and the skewness of the underlying true drift rate distribution. Our findings suggest other mechanisms are likely present in the data and cause the slow error pattern, and the effects of these mechanisms are absorbed by the random variability parameter. While the variability assumption is meaningful for theories of decision making, it should not be the only mechanism for slow errors predictions in DDM for its estimates to be meaningfully interpreted. We encourage future studies to explore mechanisms present in other evidence accumulation models, such as lateral inhibition, to better understand the mechanisms behind slow errors.

## Supplementary Information

Below is the link to the electronic supplementary material.

Supplementary Material 1

## Data Availability

The dataset is a subset of the PEERS dataset which could be publicly accessed at https://openneuro.org/datasets/ds004395/versions/2.0.0. The qualifiers for selecting the subset used in current study (participant and session lists) and processed data for the models are available at [https://osf.io/r69ws](https:/osf.io/r69ws).

## Appendix A: Priors Distributions for all Model Parameters

Each model parameter was sampled from a group-level distribution with a pre-defined mean *µ* and standard deviation *σ.* Parameters for the time lag functions were sampled from a log scale to improve sampling. Some parameters were given a low and/or a high bound for meaningful interpretation.

*Z ~* Normal_(0, inf)_(*z* ^*µ*^, *z* ^*σ*^).

*a ~* Normal_(0, inf)_(*a* ^*µ*^, *a* ^*σ*^).

*T*_*er*_ *~* Normal_(0, inf)_(*T*_*er*_^*µ*^, *T*_*er*_^*σ*^).

*S*_*t*_ *~* Normal_(0, inf)_(*S*_*t*_^*µ*^, *S*_*t*_^*σ*^).

*V*_T_ *~* Normal(*V*_T_^*µ*^, *V*_T_^*σ*^).

*V*_L_ *~* Normal(*V*_L_^*µ*^, *V*_L_^*σ*^).

*η*_T_ *~* Normal_(0, inf)_(*η*_T_^*µ*^, *η*_T_^*σ*^).

*η*_L_ *~* Normal_(0, inf)_(*η*_L_^*µ*^, *η*_L_^*σ*^).

*β*_wfT_ *~* Normal(*β*_wfT_^*µ*^, *β*_wfT_^*σ*^).

*β*_wfL_ *~* Normal(*β*_wfL_^*µ*^, *β*_wfL_^*σ*^).

*β*_*recall*_ *~* Normal_(0, inf)_(*β*_recall_^*µ*^, *β*_recall_^*σ*^).

log(*α*_recalled_) *~* Normal(log(*α*_recalled_)^*µ*^, log(*α*_recalled_)^*σ*^).

log(*k*_recalled_) *~* Normal(log(*k*_recalled_)^*µ*^, log(*k*_recalled_)^*σ*^).

log(*α*_unrecalled_) *~* Normal(log(*α*_unrecalled_)^*µ*^, log(*α*_unrecalled_)^*σ*^).

log(*k*_unrecalled_)*~* Normal(log(*k*_unrecalled_)^*µ*^, log(*k*_unrecalled_)^*σ*^).

*β*_EEGt1_ *~* Normal(*β*_EEGt1_^*µ*^, *β*_EEGt1_^*σ*^).

*β*_EEGt2_ *~* Normal(*β*_EEGt2_^*µ*^, *β*_EEGt2_^*σ*^).

*β*_EEGt3_ *~* Normal(*β*_EEGt3_^*µ*^, *β*_EEGt3_^*σ*^).

*β*_EEGt4_ *~* Normal(*β*_EEGt4_^*µ*^, *β*_EEGt4_^*σ*^).

*β*_EEGt5_ *~* Normal(*β*_EEGt5_^*µ*^, *β*_EEGt5_^*σ*^).

For the parameters that that define the prior distribution:

*z*^*µ*^, *T*_*er*_^*µ*^ *~* Normal_(0, inf)_(0.5, 0.5).

*S*_*t*_^*µ*^ *~* Normal_(0, inf)_(0.25, 0.25).

*a*^*µ*^ *~* Normal_(0, inf)_(2, 2).

*η*_T_^*µ*^, *η*_L_^*µ*^, *β*_*recall*_^*µ*^ *~* Normal_(0, inf)_(1, 1).

*V*_T_^*µ*^, *V*_L_^*µ*^ *~* Normal(2, 2).

*β*_wfT_^*µ*^, *β*_wfL_^*µ*^ *~* Normal(0, 1).

log(*α*_recalled_)^*µ*^, log(*k*_recalled_)^*µ*^, log(*α*_unrecalled_)^*µ*^, log(*k*_unrecalled_)^*µ*^ ~ Normal(0, 10).

*β*_EEGt1_^*µ*^, *β*_EEGt2_^*µ*^, *β*_EEGt3_^*µ*^, *β*_EEGt4_^*µ*^, *β*_EEGt5_^*µ*^ ~ Normal(0, 0.5).

*z*^*σ*^, *T*_*er*_^*σ*^, *S*_*t*_^*σ*^ *~* Gamma(1, 1/3).

*a*^*σ*^, η_T_^σ^, η_L_^σ^, *β*_recall_^*σ*^, *V*_T_^*σ*^, *V*_L_^*σ*^, log(*α*_recalled_)^*σ*^, log(*k*_recalled_)^*σ*^, log(*α*_unrecalled_)^*σ*^, log(*k*_unrecalled_)^*σ*^, *β*_EEGt1_^*σ*^, *β*_EEGt2_^*σ*^, *β*_EEGt3_^*σ*^, *β*_EEGt4_^*σ*^, *β*_EEGt5_^*σ*^ *~* Gamma(1, 1).

## Appendix B: Detailed Model Fitting Procedures

DE-MCMC sampling was conducted using Python code written by the last author. For each model, we set the number of chains to be equal to three times the number of parameters. We discarded the first 20,000 burn-in iterations and collected 1,000 samples from a total of 20,000 post burn-in iterations (one in every twenty iterations for thinning). We set the migration process across chains to start at 4,000th and end at 7,000th iteration for every 25 iterations during the burn-in period. For models with chains that did not converge, we continued to run the model with parameters from the latest iteration; we discarded the first 5,000 iterations and run another 20,000 iterations with the same thinning as described above.

## Appendix C: Recovered Drift Rate Parameters

**Table 3:** Recovery of drift rate variability parameters across conditions

Bias	Drift rate variability	Drift rate	Parent distribution (Lure/target)		
			Positive skewness	Normally distributed	Negative skewness
Bias to ‘old’ response	Low	High	0.30/0.27	0.32/0.71	0.24/0.71
		Medium	0.07/0.50	0.16/0.68	0.22/0.78
		Low	0.08/0.53	0.25/0.68	0.11/0.70
	High	High	0.72/1.62	0.96/2.27	1.06/2.75
		Medium	0.79/1.69	0.92/2.29	0.94/2.68
		Low	1.1 2.07	0.79/2.19	0.46/2.42
No Bias	Low	High	0.14/0.08	0.39/0.71	0.16/0.72
		Medium	0.16/0.52	0.26/0.68	0.11/0.75
		Low	0.26/0.61	0.22/0.68	0.10/0.69
	High	High	0.73/1.54	0.96/2.24	0.81/2.68
		Medium	0.81/1.73	0.92/2.28	0.92/2.58
		Low	1.1/2.26	0.87/2.29	0.52/2.24
Bias to ‘new’ response	Low	High	0.19/0.45	0.10/0.73	0.11/0.74
		Medium	0.07/0.52	0.14/0.71	0.21/0.76
		Low	0.22/0.60	0.25/0.69	0.19/0.65
	High	High	0.77/1.57	0.92/2.19	1.04/2.68
		Medium	0.75/1.78	0.96/2.3	0.95/2.34
		Low	1.09/2.45	0.90/2.24	0.57/2.0

Values calculated as recovery parameter value – parent parameter value. Bias to ‘old’ response: Z =.65. No Bias: Z =.5. Bias to ‘new’ response: Z =.35. Low drift rate variability:η_T_=.8, η_L_=.3. High drift rate variability: η_T_= 2.4, η_L_= 1. Low drift rate: V= 1.5, V_L_= 1. Medium drift rate: V_T_= 3, V_L_= 2.5. High drift rate: V_T_= 4.5, V_L_= 4. Other DDM parameters kept constant: a = 1.5, T_er_=.49, S_t_=.23.

## Appendix D: Posterior Parameter Estimates

**Table 4:** Mean group-level posterior parameter estimates across models

Models	*z*	*a*	*T*_*er*_	*S*_*t*_	*V*_T_	*V*_L_	*η*_T_	*η*_*L*_	*β*_wfT_	*β*_wfL_	*β*_recall_	*α*_recalled_
Random	0.62	1.60	0.50	0.26	2.44	1.96	2.55	0.65				
Exogenous	0.62	1.61	0.50	0.25	1.50	2.15	2.19	0.64	-0.11	-0.08	1.63	1.59
Endogenous	0.59	1.65	0.49	0.25	2.46	1.88	2.56	0.64				
Full	0.59	1.66	0.49	0.25	1.81	2.07	2.26	0.63	-0.10	-0.08	1.37	0.33

Models	*k*_recalled_	*α*_unrecalled_	*k*_unrecalled_	*β*_EEGT*1*_	*β*_EEGT2_	*β*_EEGT3_	*β*_EEGTA_	*β*_EEGL1_	*β*_EEGL2_	*β*_EEGL3_	*β*_EEGLA_
Random											
Exogenous	0.02	5.09	0.01								
Endogenous				0.29	0.43	0.57	0.16	0.05	-0.15	0.15	-0.01
Full	0.26	0.35	0.26	0.25	0.40	0.52	0.14	0.05	-0.14	0.14	-0.01

The drift rate variability for target (ηT) was a ratio parameter compared to lure variability (ηL). The drift rate parameters, VT and VL become the intercept of the regression model when drift rates were informed by regressors. Hence, they are not directly comparable with the estimates in the random model
