# Supplementary material for: Can Systematic Drift Rate Variability Replace Random Variability in the Diffusion Decision Model?
Source: Comput Brain Behav. 2026 Mar 19;9(2):261–80. doi: 10.1007/s42113-026-00264-0 (PMC13293026; doi:10.1007/s42113-026-00264-0)
Supplement: Supplementary file 1 — Supplementary Material 1 [file 42113_2026_264_MOESM1_ESM.docx]

**Supplementary Material For:**

**Can Systematic Drift Rate Variability Replace Random Variability in the Diffusion Decision Model?**

Jie Sun, Daniel Feuerriegel, Adam F. Osth

Melbourne School of Psychological Sciences, The University of Melbourne

**Corresponding Author:**

Jie Sun

[Jies3@student.unimelb.edu.au](mailto:Jies3@student.unimelb.edu.au)

Melbourne School of Psychological Sciences, Redmond Barry Building, The University of Melbourne, 3010, Australia.

**Diffusion Decision Model (DDM) Parameter Recovery with Log-Normal Across-Trial Drift Rate Distributions**

As this study is the first to examine the effect of skewness in the underlying across-trial drift rate distribution on the recovery of the *η* parameter in the DDM, it is crucial to determine whether assuming a skewed distribution has practical benefits for estimating across-trial drift rate variability in recognition memory tasks. In this study, we fit a version of the DDM that assumes across-trial drift rates are distributed as a shifted log-normal distribution. The log-normal distribution is the exponential form of a normal distribution which is positively skewed by definition.

Since the DDM with a log-normal across-trial drift rate distribution does not have a closed-form likelihood for model fitting, we applied the trapezoidal rule to approximate the likelihood under the log-normal distribution. The trapezoidal rule, a numerical integration method, approximates the area under a curve by summing the areas of smaller trapezoids. The approximation is given by the following formula:

$\int_{b}^{a} f\left( x \right)dx\approx\frac{\Delta x}{2}$ $\left( f(x_{0})+2f(x_{1} \right)+2f\left( x_{2} \right)+\ldots+2f\left( x_{N-1} \right)+f(x_{N}))$

Where *a* and *b* represent the range of values to be partitioned, $\Delta x$ is the width of each trapezoid interval equally divided by *N* number of points. The approximation is more accurate for an increasing number of trapezoids assumed (i.e., more partitions). Notably, this approximation approach has also been adopted by Smith (2023) for estimating drift rate distributions. Given that the shifted log-normal distribution extends to infinity, it was necessary to define both the range and the number of points for the approximation. We determined these values by first approximating a normal distribution of drift rates and comparing the true likelihood with the approximated likelihood. By testing different ranges (in standard deviations) and numbers of points for the approximation, we found that using 3 standard deviations and 20 points provided a robust approximation across a range of DDM parameter values without being overly computationally expensive.

To implement this method, we defined the approximation range based on the normal distribution (± 3 standard deviations around the mean) and then exponentially transformed the lowest and highest values. We selected 20 evenly spaced points from this exponential space to perform the numerical integration. For each drift rate value, we calculated point likelihoods using the DDM likelihood function without the *η* parameter and rescaled these likelihoods into the log-normal space based on the current distribution parameter values. The shape of the shifted log-normal distribution was controlled by three parameters: the mean of normal distribution *µ* before the exponential transform, the standard deviation of the normal distribution *σ*, and a shift parameter *d* that moves the log-normal distribution horizontally. Finally, these likelihood values were used to apply the trapezoidal rule for approximation. We assumed that both target and lure drift rate distributions are positively skewed, corresponding to similar distributions for memory strength across trials.

For the log-normal distribution, the standard deviation of the normal distribution before exponentiation controls the variance and the skewness of the log-normal distribution. The greater the standard deviation, the greater the skewness and variance of the log-normal distribution. We fitted the DDM with log-normal distributed drift rates to the recognition memory dataset. However, we observed that the estimate of the standard deviation parameter was very small, especially for targets, and therefore the resulting log-normal distribution had small variance and a shape that approximated the normal distribution (see Table 1). This means that we observed little evidence for a positively skewed drift rate distribution assumption in the DDM for the data collected during the recognition memory task.

**Supplementary Table 1.**

Estimated group-level log-normal distribution parameters for targets and lures, and calculated variance and skewness.

|  | Targets | Lures |
| --- | --- | --- |
| *µ* | 1.05 (-.11, 1.61) | .05 (-.65, .37) |
| *σ* | .03 (.00, .10) | .59 (.58, .60) |
| *d* | -.36 (-2.50, 1.51) | -2.77 (-3.57, -2.05) |
| SD | .09 | .81 |
| Skewness | .09 | 2.2 |
| Note. SD $=\sqrt{{(e}^{\sigma^{2}}-1)e^{(2\mu+ \sigma^{2})}}$, Skewness = ${(e}^{\sigma^{2}}+2)\sqrt{e^{\left( \sigma^{2} \right)}-1}.$ Calculations were based on most likely parameter values. Numbers in the parentheses indicate 95% highest density interval from the posterior distribution. | | |

**Slow Error Predictions by The Models**


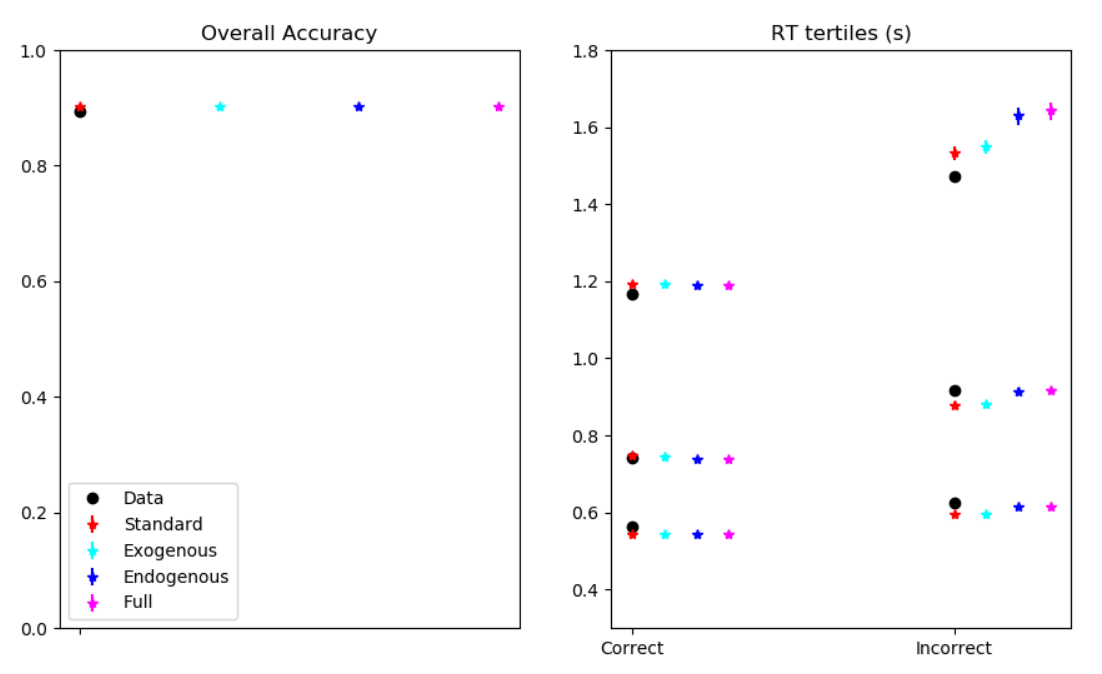


Supplementary Figure 1. Model predictions of overall accuracy and RT tertiles (10%, 50%, 90%) across models.

**Effects of Additional Unknown Sources of Slow Errors**

While previous studies have suggested that the estimations of the *η* parameter were driven by the presence of slow errors (Yap et al., 2012), it is important to directly demonstrate this property of DDM. Here, we performed additional parameter recovery practices based on the simulated data. While the slow errors in the original simulated data was solely caused by the drift rate variability, we aim to understand how the variability estimates will change if additional unknown sources of slow errors were present in the data.

To test this idea, we added a positively skewed distribution to the RTs of error trials in the simulation, causing the errors to be even slower. We then fitted the Random, Exogenous and Full models to this data. Overall, we observed inflated estimates of the overall drift rate variability across models (SFig. 2). This was expected as the model relies on this mechanism to account for slow errors.


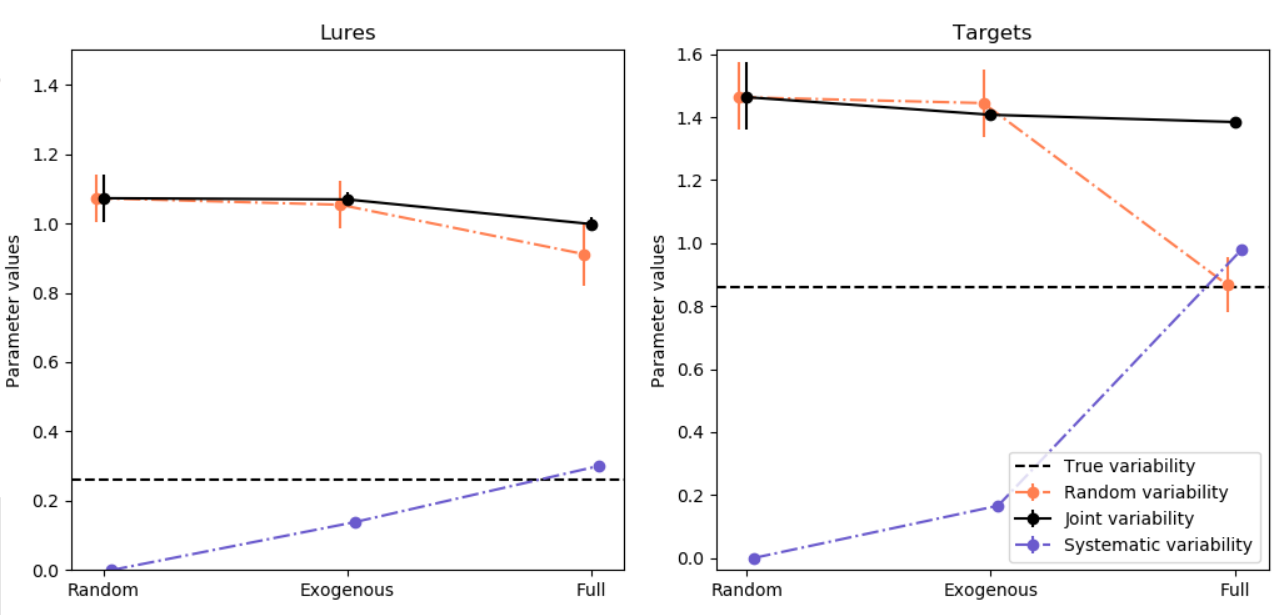


Supplementary Figure 2. Recovery of variability estimates based on simulated data with additional unknown source of slow errors. The error bars represent the 95% highest posterior density interval. Random = standard DDM, Exogenous = DDM with exogenous factors, Full = DDM with both exogenous and endogenous factors. The vertical dashed lines indicate the true variabilities in the simulation.

**References**

Smith, P. L. (2023). “Reliable organisms from unreliable components” revisited: the linear drift, linear infinitesimal variance model of decision making. *Psychonomic bulletin & review*, *30*(4), 1323-1359.

Yap, M. J., Balota, D. A., Sibley, D. E., & Ratcliff, R. (2012). Individual differences in visual word recognition: insights from the English Lexicon Project. *Journal of Experimental Psychology: Human Perception and Performance*, *38*(1), 53.
